# Comparison of Titanium and Carbon Fiber-Reinforced Polyetheretherketone Spinal Implants in Postoperative Spine Stereotactic Body Radiation Therapy: A Comprehensive Review of Current Evidence and Future Perspectives

**DOI:** 10.1016/j.adro.2026.102136

**Published:** 2026-07-04

**Authors:** Yuhi Suda, Yujiro Nakajima, Kei Ito, Shota Watanabe, Kentaro Taguchi, Satoshi Kito

**Affiliations:** a Division of Radiation Oncology, Department of Radiology, Tokyo Metropolitan Cancer and Infectious Diseases Center Komagome Hospital, Bunkyo-ku, Tokyo, Japan; b Department of Radiological Sciences, Komazawa University, Setagaya-ku, Tokyo, Japan

## Abstract

Postoperative stereotactic body radiation therapy (SBRT) for spinal metastases presents technical challenges, particularly in the presence of internal fixation devices. Titanium implants—the standard implant material—cause substantial imaging artifacts in computed tomography (CT) and magnetic resonance (MR) scans, which can lead to target and organ-at-risk contouring problems and compromise the accuracy of dose calculations. Meanwhile, carbon fiber-reinforced polyetheretherketone (Carbon/PEEK) implants have emerged as radiolucent alternatives with favorable imaging properties. This review summarizes the current evidence comparing titanium and Carbon/PEEK implants in the context of postoperative spine SBRT, focusing on differences in artifact size in CT and MR imaging, contouring accuracy, dose perturbation, dose calculation accuracy, image guided radiation therapy, radiation therapy robustness to setup error, and clinical outcomes in photon and proton therapies.

## Introduction

Metastatic epidural spinal cord compression, which occurs in 10% of patients with cancer,^1^^,^^2^ is a serious complication of spinal metastases, presenting with pain, paralysis, sensory loss, and bladder or bowel dysfunction.^3^ A randomized trial showed that surgical decompression followed by 30 Gy in 10 fractions of conventional external beam radiation therapy (cEBRT) improved ambulation compared with cEBRT alone.^4^ In surgery, the lamina is removed to relieve pressure, and the resulting spinal instability necessitates internal fixation with rods and screws.^5^

The titanium alloy Ti6Al4V is widely regarded as the standard material for implants in metastatic spinal tumor surgery owing to its exceptional corrosion resistance, favorable strength-to-weight ratio, and high fatigue strength.^6^ However, titanium causes artifacts in computed tomography (CT) and magnetic resonance (MR) scans, which can affect the contouring of tumors and surrounding organs, along with dose calculations. In cEBRT, a simple field delivers a uniform, low dose to the entire region, including the spinal cord; therefore, metal artifacts rarely cause serious problems.

Recently, stereotactic body radiation therapy (SBRT) has been widely recognized as a treatment option following decompression surgery for spinal cord compression.^7^ SBRT enables the delivery of ablative radiation doses to the tumor using an ultrahypofractionated regimen, achieving excellent local control (LC).^8^ Spine SBRT requires accurate contouring, steep dose gradients, and precise positioning because the target and important serial organs, such as the spinal cord, are always adjacent. However, artifacts induced by titanium implants may lead to several problems in the setting of postoperative SBRT. The problems include the imprecise contouring of the target and spinal cord, dose perturbation and uncertainty in dose calculations, and image guided radiation therapy (IGRT) and radiation therapy robustness issues. Any compromise in treatment accuracy resulting from these issues significantly increases the risk of severe normal tissue injury, such as radiation myelopathy, particularly in the context of spine SBRT, where ablative doses are delivered in close proximity to the spinal cord.^9^^,^^10^

**Figure 1:**
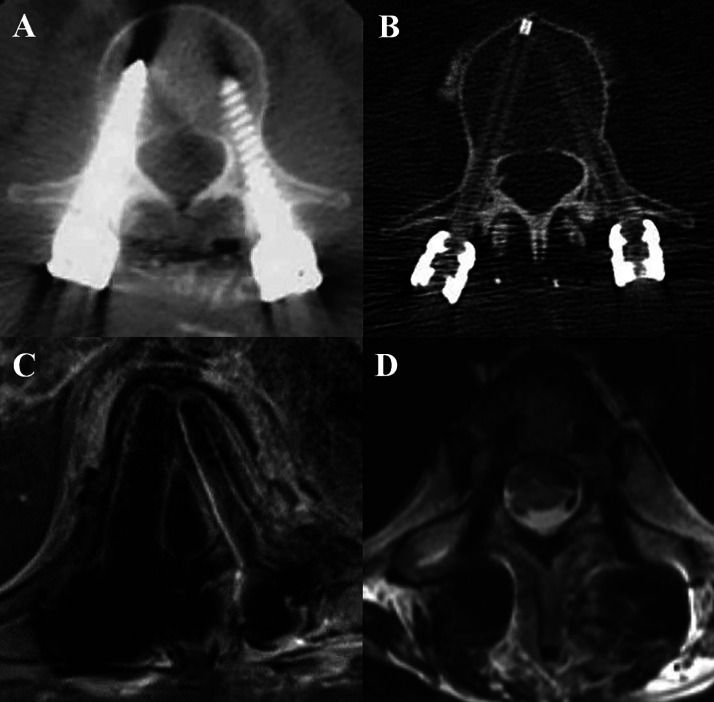
Comparison of image quality between titanium and Carbon/PEEK implant. (A) Axial CT image of a titanium implant. (B) CT image of a hybrid Carbon/PEEK implant. (C) Axial MR image of a titanium implant. (D) MR image of a hybrid Carbon/PEEK implant. *Abbreviations*: Carbon/PEEK = carbon fiber-reinforced polyetheretherketone; CT = computed tomography; MR = magnetic resonance.

Carbon fiber-reinforced polyetheretherketone (Carbon/PEEK) has been used in spinal implants in recent years. This material comprises a PEEK with an elastic modulus of 3.6 GPa, which is close to that of cortical bone (17-21 GPa),^11^ mixed with carbon fibers to increase the device strength. In addition, Carbon/PEEK is radiolucent; thus, it has the advantage of producing images with fewer artifacts and therefore fewer dose perturbations than titanium (Fig. 1). Although they also have the disadvantage of higher costs compared with titanium implants, a comprehensive assessment of their potential benefits and limitations is warranted.^12^^,^^13^

The aim of this review is to clarify the potential advantages and limitations of Carbon/PEEK implants by comparing them with titanium implants in terms of the artifact sizes in CT and MR imaging, contouring accuracy, dose perturbation, dose calculation accuracy, IGRT, radiation therapy robustness, and clinical outcomes.

## Methods and Materials

This narrative review was conducted in accordance with the Scale for the Assessment of Narrative Review Articles guidelines.^14^ PubMed and Scopus were searched for records published up to April 2026. The search was performed using various combinations of the keywords “carbon,” “CFR,” “titanium,” “spine SBRT,” “radiotherapy,” “dose calculation,” “imaging artifact,” “spine implant,” and “spinal implant” to ensure comprehensive retrieval of relevant articles. The search included peer-reviewed studies published in English, without restrictions on publication date. This approach was intended to capture the full spectrum of available evidence regarding comparisons between titanium and Carbon/PEEK implants, ranging from foundational studies to recent clinical advances.

### Artifacts in imaging

#### Artifacts in CT imaging

The artifact sizes in CT imaging of titanium and Carbon/PEEK implants have been compared in several studies (Table 1). These studies consistently demonstrated that Carbon/PEEK implants have significantly fewer metal artifacts and smaller Hounsfield unit (HU) fluctuation than titanium implants. Poel et al^15^ reported that the artifact size was reduced by 50% for hybrid Carbon/PEEK implants (Carbon/PEEK with a titanium tulip head) and by 90% for Carbon/PEEK compared with titanium implants in a torso phantom. A small-scale patient study also showed reduced artifacts with Carbon/PEEK, demonstrating that hybrid Carbon/PEEK implants substantially reduced artifacts by approximately 50% or more compared with titanium implants.^16^^,^^17^ Furthermore, Kratzig et al^18^ demonstrated significant differences in the HU value fluctuation of a gel phantom in the anterior and posterior regions of the vertebral body and spinal canal, with a particularly large difference in the anterior region of the vertebral body (titanium: 201.2 ± 42.7 HU vs Carbon/PEEK: 70.4 ± 28.4 HU, *P* < .001). Even research involving patients and cadavers has indicated that HU value fluctuations can be reduced with Carbon/PEEK compared with titanium implants.^19^^,^^20^

**Table 1:** Comparison of metal artifacts between titanium and Carbon/PEEK implants in CT imaging

Reference	Year	Object	MAR	Summary of results
Poel et al^15^	2020	Torso phantom	n/a	Carbon/PEEK implants reduced artifact volume (titanium: 390.6 cc vs hybrid Carbon/PEEK: 174.2 cc vs Carbon/PEEK: 33.9 cc). Carbon/PEEK implants reduced artifact area overlapped with GTV (titanium: 58.2% vs hybrid Carbon/PEEK: 0% vs Carbon/PEEK: 0%).
Kumar et al^16^	2024	Titanium: 42, Carbon/PEEK: 20 patients	n/a	Carbon/PEEK implants significantly reduced artifact volume (titanium: 73.4 ± 50.43 mm^3^ vs Carbon/PEEK: 20.0 ± 20.7 mm^3^, *P* < .001).
Ringel et al^17^	2017	13 patients each	n/a	Carbon/PEEK implants significantly reduced artifact volume (titanium: 937 ± 322 mm^3^ vs Carbon/PEEK: 494 ± 246 mm^3^, *P* < .001).
Kratzig et al^18^	2021	Gel phantom	iMAR	Carbon/PEEK implants significantly reduced HU value fluctuation at the spinal canal (titanium: −30.7 ± 108.8 HU vs Carbon/PEEK: 33.4 ± 34.6 HU, *P* < .001), at the posterior of the vertebral body (titanium: 68.9 ± 69.92 HU vs Carbon/PEEK: 59.8 ± 22.34 HU, *P* < .001), and at the anterior of the vertebral body (titanium: 201.2 ± 42.7 HU vs Carbon/PEEK: 70.4 ± 28.4 HU, *P* < .001).
Rijs et al^19^	2024	11 patients each	n/a	Carbon/PEEK implants reduced postoperative HU value fluctuation compared to preoperative at the spinal cord compression level (titanium: 2.51‑fold vs Carbon/PEEK 1.43‑fold).
Kalasauskas et al^20^	2022	92 screws in a cadaver	n/a	Carbon/PEEK implants reduced HU value fluctuation (titanium: 78 ± 254 HU vs hybrid Carbon/PEEK: 60 ± 147 HU vs Carbon/PEEK: 30 ± 124 HU).
Huber et al^24^	2021	6 sheep spine cadavers	iMAR, DECT, Reconstruction kernel	Carbon/PEEK implants reduced the artifacts compared to using MAR with titanium implants.
Ho et al^25^	2024	Spinal phantom, patient	SEMAR	Carbon/PEEK implants significantly reduced the artifact index at the vertebral canal (titanium: 66.76 vs Carbon/PEEK: 6.55, *P* = .005), with similar results around the screw.
Shi et al^26^	2022	Torso phantom	iMAR	Carbon/PEEK implants significantly reduced artifact volumes (titanium: 77.46 cc vs hybrid Carbon/PEEK: 9.54 cc vs Carbon/PEEK: 0.50 cc). Carbon/PEEK implants reduced HU value fluctuation at the spinal canal (titanium: 63.8 HU vs Carbon/PEEK: 6.1 HU).

*Abbreviations:* Carbon/PEEK = carbon fiber-reinforced polyetheretherketone; CT = computed tomography; DECT = dual-energy computed tomography; GTV = gross tumor volume; HU = Hounsfield unit; hybrid Carbon/PEEK = Carbon/PEEK with titanium tulip head; iMAR = iterative metal artifact reduction; MAR = metal artifact reduction; n/a = not applicable; SEMAR = single-energy metal artifact reduction.

For metal implants, metal artifact reduction (MAR) techniques can be used to reduce artifacts, with MAR algorithm reconstruction and dual-energy CT being the main methods employed.^21^, ^22^, ^23^ It has been reported that Carbon/PEEK implants reduce artifacts more effectively than titanium implants, even when using MAR techniques with titanium.^18^^,^^24^, ^25^, ^26^

#### Artifacts in MR imaging

The artifact sizes of titanium and Carbon/PEEK implants in MR imaging have been compared in several studies (Table 2). Kratzig et al^18^ reported fewer artifacts with Carbon/PEEK implants than with titanium implants, as assessed visually using a gel phantom. Patient and cadaver studies also indicated that Carbon/PEEK implants reduced artifacts compared with titanium implants.^17^^,^^20^^,^^27^^,^^28^ Almeida et al^27^ reported that Carbon/PEEK implants improved 3 parameters related to artifacts compared with titanium implants: mean pedicle screw artifact size (titanium: 13.2 mm vs Carbon/PEEK: 5.8 mm), canal visualization (titanium: 15.5 mm vs Carbon/PEEK: 19.2 mm), and sagittal distortion (titanium: 1.9 mm vs Carbon/PEEK: 0.5 mm), with *P* < .001 in all cases. Furthermore, Fleege et al^28^ demonstrated that Carbon/PEEK implants significantly increased the artifact-free vertebral body area compared with titanium implants (titanium: 48.3% vs Carbon/PEEK: 67.1%, *P* ≤ .01). Qualitative analysis revealed severe artifacts hampering the evaluation at the spinal canal after placing implants (titanium: 28% vs hybrid Carbon/PEEK: 2% vs Carbon/PEEK: 0%, *P* < .001).^20^ In another study, the assessability of the lumbar spine was significantly improved with Carbon/PEEK screws for all measurements (*P* ≤ .01).^28^ In addition, Carbon/PEEK implants significantly reduced the artifact compared with titanium implants with different screw sizes: small (titanium: 12.6 mm vs Carbon/PEEK: 5.7 mm, *P* < .001), medium (titanium: 13.2 mm vs Carbon/PEEK: 6.5 mm, *P* < .001), and large (titanium: 15.3 mm vs Carbon/PEEK: 6.1 mm, *P* < .001).^27^ MAR techniques have also been applied to MR scans to reduce metal artifacts,^29^ but this often extends the imaging time.^30^ In addition, it has been reported that Carbon/PEEK reduced artifacts and enhanced visualization compared with titanium in various sequences that included MAR.^18^ These investigations have consistently demonstrated that Carbon/PEEK implants reduce artifacts compared with titanium implants in MR imaging.

**Table 2:** Comparison of metal artifacts between titanium and Carbon/PEEK implants in MR imaging

Reference	Year	Object	Magnetic field strength	Summary of results
Ringel et al^17^	2017	17 patients each	1.5 T, 3 T	Carbon/PEEK implants reduced artifacts compared with using MAR with titanium implants in the postoperative image.
Kratzig et al^18^	2021	Gel phantom	3 T	Carbon/PEEK implants exhibited little change in the ROI intensity and improved visualization in all sequences, including MAR.
Kalasauskas et al^20^	2022	92 screws in cadaver	1.5 T, 3 T	Titanium implants caused artifacts, significantly hampering the evaluation of the spinal canal in qualitative analysis (titanium: 28% vs hybrid Carbon/PEEK: 2% vs Carbon/PEEK: 0%, *P* < .001).
Almeida et al^27^	2024	Four patients each	1.5 T	Carbon/PEEK implants significantly improved mean artifact size at the pedicle screw (titanium: 13.2 mm vs Carbon/PEEK: 5.8 mm), canal visualization (titanium: 15.5 mm vs Carbon/PEEK: 19.2 mm), and sagittal distortion (titanium: 1.9 mm vs Carbon/PEEK: 0.5 mm), all *P* < .001.
Fleege et al^28^	2020	Four patients each	1.5 T	Carbon/PEEK significantly increased the artifact-free vertebral body area compared with titanium (titanium: 48.3% vs Carbon/PEEK: 67.1%, *P* ≤ .01). Assessability of the lumbar spine was significantly improved for Carbon/PEEK screws (*P* ≤ .01) for all measurements.

*Abbreviations:* Carbon/PEEK = carbon fiber-reinforced polyetheretherketone; hybrid Carbon/PEEK = Carbon/PEEK with a titanium tip head; MAR = metal artifact reduction; MR = magnetic resonance; ROI = region of interest; T = Tesla.

#### Artifacts in positron emission tomography/CT

Positron emission tomography (PET)/CT is widely used for cancer diagnosis, staging, treatment planning, and posttreatment response assessment. However, PET images are typically reconstructed with attenuation correction based on CT data. Therefore, when metallic artifacts are present in the CT images, attenuation correction errors may lead to false uptake artifacts around the metal on the PET images. In fact, titanium implants have been reported to cause such false uptake due to metal-induced artifacts, which can make clinical image interpretation difficult, particularly in the spine and pelvis.^31^ MAR algorithms in CT are considered beneficial for PET/CT because they reduce metal artifacts. For example, a study of patients with hip prostheses demonstrated that MAR significantly improved the quantitative accuracy of standardized uptake values.^32^^,^^33^ Although data specific to spinal implants remain limited, an evaluation of 100 patients, including individuals with spinal hardware, showed that while iterative MAR improved CT numbers, it did not result in significant differences in standardized uptake value measurements.^34^ Consistent with findings from CT imaging studies, in which Carbon/PEEK implants reduced artifacts more effectively than titanium implants even when MAR algorithms were applied,^18^^,^^24^, ^25^, ^26^ the selection of Carbon/PEEK implants may be more effective for optimizing PET/CT image quality. These findings suggest that the use of Carbon/PEEK implants may help improve the diagnostic accuracy of PET/CT images.

### Contouring accuracy for radiation therapy

Deviation from contouring guidelines impacts local tumor control in spine SBRT.^35^ In patients who have received titanium implants in spine surgery, contouring uncertainties increase owing to the influence of artifacts in CT and MR images. Kovacs et al^23^ compared the contouring accuracy in phantoms without implants and titanium implants with or without MAR and found that both the Hausdorff distance (without implant: 2.2 mm vs titanium: 18.2 mm vs titanium with MAR: 18.6 mm) and dice coefficient (without implant: 0.88 vs titanium: 0.57 vs titanium with MAR: 0.74) worsened with titanium implants, and the enhancement with MAR remained limited. Although Carbon/PEEK implants are expected to provide higher contouring accuracy, there are no reports directly comparing the contouring accuracy of Carbon/PEEK and titanium for CT and MR images. Therefore, additional studies are required to determine contour accuracy.

### Dose perturbation and dose calculation accuracy

#### Photon therapy

When radiation interferes with metal implants, perturbations can cause hot and cold spots to appear in the dose distribution.^36^ In addition, dose calculations using a radiation therapy planning system (RTPS) in cases with metal implants can be affected by large uncertainties owing to the metal artifacts.^22^ Titanium and Carbon/PEEK implants have been compared in terms of perturbations and dose calculation accuracy in photon therapy (Table 3). When photons or electrons pass through a metal implant, secondary electrons or scattering can cause dose perturbation, resulting in an overdose on the front surface of the metal and tissue and a lower dose on the rear surface.^36^^,^^37^ Large perturbations are observed for titanium implants, but this effect can be reduced by using Carbon/PEEK implants.^25^^,^^38^ Ho et al^25^ showed that dose attenuation by titanium implants was observable across all profiles and minimal dose attenuation by the Carbon/PEEK in a slab phantom. In addition, a comparison of 6-MV single photon beams using Monte Carlo calculations revealed that the maximum perturbation for titanium is 30%, compared with 5% for Carbon/PEEK.^39^

**Table 3:** Comparison of the perturbations and dose calculation accuracy for titanium and Carbon/PEEK implants in photon therapy

Reference	Year	Object	Evaluation item	Evaluation equipment	Summary of results
Ho et al^25^	2024	Slab phantom	Perturbation and dose calculation	Gafchromic film	Carbon/PEEK implants improved the dose calculation accuracy at 2 mm behind the screw (titanium: 2.88% vs Carbon/PEEK: 2.07%, *P* < .001), and at the longitudinal axis (titanium: 3.35% vs Carbon/PEEK: 1.34%, *P* < .001).
Shi et al^26^	2022	Torso phantom	Perturbation	RTPS	Plan quality showed no significant difference between the groups without implant, titanium implant, and Carbon/PEEK implant.
Nevelsky et al^39^	2017	Water	Perturbation	Monte Carlo	Carbon/PEEK implants reduced the maximum dose attenuation of a single photon beam (titanium: >30% vs Carbon/PEEK: <5%).
Muller et al^44^	2020	10 patients	Perturbation	RTPS	When a 6% uncertainty was applied to the density override, the recalculated VMAT dose distributions and DVH changes in the criteria were slightly larger for titanium, but all deviations were <1%.
Sahinoglu et al^45^	2026	Sheep cadaver	Dose calculation	TLD	Titanium screws and rods caused a dose difference of approximately 14% on their surface; this discrepancy was reduced to only 2.2% on the rods when Carbon/PEEK implants were used.
Goodall et al^46^	2025	Lumber spinal phantom	Dose calculation	Ionization chambers, Gafchromic film	Dose deviation within the PTV reached 5%-6% for titanium implants without MAR, whereas it remained within 2%-3% for Carbon/PEEK implants.
Prochazka et al^47^	2026	Porcine vertebral specimen	Dose calculation	Gafchromic film	Carbon/PEEK implants achieved calculation accuracy comparable with that without implant, whereas titanium caused the maximum dose deviation (GPR of 10-MV FFF at vertebral body; without implant: 93.5% vs titanium: 88.3% vs Carbon/PEEK: 96.5%).

*Abbreviations:* Carbon/PEEK = carbon fiber-reinforced polyetheretherketone; DVH = dose-volume histogram; FFF = flattening filter-free; GPR = gamma pass rate; PTV = planning target volume RTPS = radiation therapy treatment planning system; TLD = thermoluminescent dosimeters; VMAT = volumetric modulated arc therapy.

The dose difference between measurement and RTPS calculation was significantly smaller for Carbon/PEEK than for titanium implants in a slab phantom with a single rectangular beam, 2 mm behind the screw at the tulip head (titanium: 2.88% vs Carbon/PEEK: 2.07%, *P* < .001) and along the longitudinal axis (titanium: 3.35% vs Carbon/PEEK: 1.34%, *P* < .001).^25^ In spine SBRT, techniques such as volumetric modulated arc therapy (VMAT), which delivers radiation continuously from a rotating gantry to achieve highly multidirectional irradiation, can distribute and thereby mitigate the effects of dose perturbation and calculation uncertainties.^26^ Considering titanium implants, Son et al^40^ compared dose calculations and measurements across different linacs and target sizes using an ionization chamber, finding that the 2 agreed within 3% under all conditions; however, the average dose was still 1.9% lower than that without implants, a statistically significant difference (*P* < .01). Cheng et al^41^ reported that in the case of film measurements with 3%/3 mm criteria (3% dose difference and 3 mm distance-to-agreement), there were unacceptable differences with a gamma pass rate (GPR) of <90% in the shadow of the titanium implant when a single beam was used, but when using 9-field intensity-modulated radiation therapy (IMRT), the GPR was improved to >95%. In a multicenter study, Furuya et al^42^ measured the target dose using radiophotoluminescent glass dosimeters and found that the results were within 5% of the calculated dose.

In addition, studies have suggested that the presence or absence of MAR for CT, differences in calculation algorithms, and titanium density overrides have little effect on dose calculations.^26^^,^^41^^,^^43^ When a 6% uncertainty override was applied to titanium HU values, the effect was negligible and smaller than 1%, similar to Carbon/PEEK.^44^ However, the RTPS algorithm may lead to dose differences exceeding 10% for both the tumor and the spinal cord under different conditions.^43^ For Carbon/PEEK implants, evaluation was performed using film measurements with CyberKnife (Accuray), and a GPR of ≥97% was achieved with a 1 mm/5% tolerance.^38^ Recently, several studies have directly compared dose calculation accuracy between titanium and Carbon/PEEK implants using IMRT techniques. In an evaluation of multifield IMRT using thermoluminescent dosimeters in a sheep cadaver model, titanium screws and rods caused a dose difference of approximately 14% at the implant surface, whereas this discrepancy was reduced to only 2.2% at the rods when Carbon/PEEK implants were used.^45^ In a VMAT study, Goodall et al^46^ demonstrated using film dosimetry that dose deviation within the planning target volume reached 5% to 6% for titanium implants without MAR, whereas it remained within 2% to 3% for Carbon/PEEK implants. Furthermore, Prochazka et al^47^ evaluated the GPR of a VMAT plan using film dosimetry and reported that Carbon/PEEK implants achieved calculation accuracy comparable withthat observed without implants, whereas titanium implants produced the greatest dose deviation (10-MV flattening filter-free at the vertebral body: without implant, 93.5% vs titanium, 88.3% vs Carbon/PEEK, 96.5%).

These recent studies clearly demonstrate that Carbon/PEEK implants significantly reduce dose perturbations and improve calculation accuracy compared with titanium implants in photon-based spine SBRT. These findings suggest that clinical implementation of Carbon/PEEK implants may substantially improve the dosimetric reliability and precision of postoperative radiation therapy.

#### Proton therapy

Considering titanium implants in proton therapy, the shift of the Bragg peak may result in far greater dose reduction to the target compared with the photon beam.^17^ The perturbation and dose calculation accuracy between titanium and Carbon/PEEK implants in proton therapy have been reported (Table 4). The film measurement results for a single proton beam behind the screw indicated differences in the dose perturbations at 0 cm (titanium: caused dose perturbation vs Carbon/PEEK: not significant), 2 cm (titanium: significant dose enhancement at the screw edges vs Carbon/PEEK: not significant), and 4 cm (titanium: reduction of 80% vs Carbon/PEEK: reduction of 20%); Carbon/PEEK implants caused a slight beam perturbation compared with titanium.^48^ In addition, the quality of the treatment plan was improved with Carbon/PEEK implants compared with titanium.^26^^,^^44^ Muller et al^44^ reported that dose distributions exhibited reduced dose conformity and homogeneity in proximity to the titanium implant, caused by the large density gradient of the penetrated materials. In addition, the coverage and target dose homogeneity were superior for Carbon/PEEK compared with titanium implants.^44^

**Table 4:** Comparison of the perturbations and dose calculation accuracy for titanium and Carbon/PEEK implants in proton therapy

Reference	Year	Object	Evaluation item	Evaluation equipment	Summary of results
Poel et al^15^	2020	Torso phantom	Dose calculation	Gafchromic film	Carbon/PEEK implants improved the GPR in a single beam (without implant: 83.5% vs titanium: 74.0% vs hybrid Carbon/PEEK: 67.1% vs Carbon/PEEK: 83.3%) and a clinical plan (without implant: 83.2% vs titanium: 81.3% vs hybrid Carbon/PEEK: 78.9% vs Carbon/PEEK: 85.2%).
Shi et al^26^	2022	Torso phantom	Perturbation	RTPS	Plan quality differed more for titanium implants than for the group without implants, but Carbon/PEEK implants were similar compared to those without implants.
Mastella et al^48^	2017	Slab phantom	Perturbation, dose calculation	Gafchromic film	Carbon/PEEK implants reduced dose perturbation in a single beam at 0 cm (titanium: caused dose perturbation vs Carbon/PEEK: not significant), 2 cm (titanium: significant dose enhancement at the screw edges vs Carbon/PEEK: not significant), and 4 cm (titanium: 80% of dose reduction vs Carbon/PEEK: 20% dose reduction).
Nevelsky et al^39^	2017	Water	Perturbation	Monte Carlo	Carbon/PEEK screw reduced the maximum dose perturbation (titanium: >30% vs Carbon/PEEK: <5%).
Muller et al^44^	2020	10 patients	Perturbation	RTPS	A 6% uncertainty was applied to the override of the HU values; Carbon/PEEK improved heterogeneous target coverage (titanium: 7.6 ± 2.3% vs Carbon/PEEK: 3.4 ± 1.2%).

*Abbreviations:* Carbon/PEEK = carbon fiber-reinforced polyetheretherketone; GPR = gamma pass rate; HU = Hounsfield unit; hybrid Carbon/PEEK = Carbon/PEEK with titanium tip head; RTPS = radiation therapy treatment planning system.

The uncertainty in dose calculation using an RTPS for metal implants due to the Bragg peak shift may be greater than that in photon beam therapy.^17^ Poel et al^15^ verified the GPR with film measurement using a torso phantom with a 3 mm/3% tolerance in a single-beam system (without implant: 83.5% vs titanium: 74.0% vs hybrid Carbon/PEEK: 67.1% vs Carbon/PEEK: 83.3%) and for a clinical plan (without implant: 83.2% vs titanium: 81.3% vs hybrid Carbon/PEEK: 78.9% vs Carbon/PEEK: 85.2%). The GPR decreased upon the inclusion of titanium, and hot and cold spots were observed in areas associated with titanium implants.^15^ When comparing Carbon/PEEK with titanium implants, hot spots in at-risk organs and cold spots in the tumor volume were reduced by up to 50%.^15^

It has been asserted that proton beam therapy must ensure accurate contouring of the metal hardware and appropriately apply material overrides.^49^ Poel et al^15^ demonstrated that dosimetry remains unaffected even without overrides for Carbon/PEEK implants. Muller et al^44^ reported that when a 6% uncertainty was applied to the override of the HU values, the heterogeneous target coverage measured considering the standard deviation inside the target was improved with Carbon/PEEK implants (titanium: 7.6 ± 2.3% vs Carbon/PEEK: 3.4 ± 1.2%). Furthermore, the time required for artifact contouring was reduced with Carbon/PEEK implants (titanium: 172 min vs Carbon/PEEK: 118 min).^15^

These perturbations and calculation uncertainties can be mitigated by avoiding beam paths through implants or by increasing the number of fields.^26^^,^^49^ However, spine SBRT requires the use of a posterior beam,^49^ and it is often difficult to prevent this beam from passing through the implant. In addition, an increase in the irradiation field may lead to an increase in the dose to normal tissue.^26^ For this reason, in contrast to photon therapy, the irradiation angle and number of fields are limited in proton therapy. Owing to these restrictions, the perturbation and uncertainty of dose calculations may be considerably reduced by using Carbon/PEEK implants instead of titanium implants.

### IGRT and robustness for setup error

The accuracy of patient positioning should be verified with daily IGRT before treatment delivery in spine SBRT.^49^^,^^50^ The smaller artifacts produced by Carbon/PEEK implants compared with titanium implants suggest that implant, bone, and soft tissue registration in IGRT may be more accurate with Carbon/PEEK implants. Henzen et al^38^ evaluated IGRT accuracy using the CyberKnife system and demonstrated that 2D-3D matching achieved an accuracy of 0.71 mm, which is consistent with that achieved in regular machine quality assurance, even when a hybrid Carbon/PEEK implant was present. Considering titanium implants, multicenter end-to-end trials revealed that the irradiation accuracy was within 2 mm at all facilities.^42^ A study comparing couch displacement measurements obtained using cone-beam computed tomography and stereoscopic X-ray imaging among conditions without implants, with titanium implants, and with Carbon/PEEK implants demonstrated differences within 0.3 mm under all conditions, suggesting that imaging artifacts did not substantially impair localization accuracy.^46^ Registration errors appear to be small with both titanium and Carbon/PEEK implants; however, additional direct comparative studies are required because of the limited number of available reports.

Radiation therapy robustness considering setup uncertainty has been evaluated for only proton therapy (Table 5).^15^^,^^26^ These studies have indicated that using Carbon/PEEK implants strengthens the robustness of treatment plans compared with titanium implants.^15^^,^^26^ Poel et al^15^ indicated that the use of Carbon/PEEK implants exhibits a plan robustness similar to that obtained without implants with a clinical target volume underdose error of <5% (without implant: 4.8% vs titanium: 17.3% vs hybrid Carbon/PEEK: 8.5% vs Carbon/PEEK: 4.1%). The same trend was observed for the clinical target volume overdose error and spinal cord. Hybrid Carbon/PEEK implants have also been reported to be more robust than titanium alone.^15^ These findings indicate that it is possible to develop robust treatment plans for proton therapy using these implants. Although the influence of implants on treatment robustness is assumed to be smaller in photon therapy than in proton beam therapy, this hypothesis remains unverified and warrants further investigation.

**Table 5:** Comparison of the robustness of titanium and Carbon/PEEK implants

Reference	Year	Object	Beam quality	Summary of results
Poel et al^15^	2020	Torso phantom	Proton	Carbon/PEEK implants exhibited plan robustness similar to that observed without implants, with a CTV underdose error of >5% (without implant: 4.8% vs titanium: 17.3% vs hybrid Carbon/PEEK: 8.5% vs Carbon/PEEK: 4.1%).
Shi et al^26^	2022	Torso phantom	Proton	When evaluating target coverage and OAR dose for uncertainty scenario analysis, considering the initial plan, higher mean values and narrower bands of dose variations were achieved for the groups without implants and with Carbon/PEEK implants.

*Abbreviations:* Carbon/PEEK = carbon fiber-reinforced polyetheretherketone; CTV = clinical target volume; hybrid Carbon/PEEK = Carbon/PEEK with titanium tip head; OAR = organ at risk.

### Clinical outcomes for radiation therapy

A systematic review including 326 patients from 11 articles indicated that the local recurrence rate in the Carbon/PEEK group was comparable with that in the titanium group (titanium: 10.7% over a 20.2-month mean follow-up vs Carbon/PEEK: 14.4% over a 10.7-month mean follow-up).^12^ However, this study was focused on retrospective or ambispective reports, including various radiation modalities and techniques, and it involved a significant proportion of nonbone metastases.^12^ Because of this heterogeneity, no definitive conclusions can be drawn from the study.

In photon therapy, a case-control matched analysis revealed no significant difference in local recurrence of spinal metastases between titanium and Carbon/PEEK patients (31% vs 22%, *P* = .31).^51^ Although this study included cEBRT and SBRT, SBRT requires high accuracy in terms of parameters, including contouring and dose calculations. Therefore, the impact of implant differences on clinical outcomes is expected to be greater in SBRT than in cEBRT. A retrospective study focusing exclusively on SBRT demonstrated significantly longer local progression-free survival with Carbon/PEEK implants compared with titanium implants (HR, 0.127; 95% CI, 0.017-0.945; *P* = .044).^52^ Although further investigation is warranted, Carbon/PEEK implants may offer comparable or superior therapeutic effects to titanium implants in photon therapy. To provide higher-level evidence, a prospective randomized controlled trial, the CarbonS spine trial, is currently underway to directly compare LC rates between titanium and Carbon/PEEK implants in patients undergoing postoperative spine SBRT. The results of this and other ongoing studies are expected to provide more definitive conclusions regarding the clinical superiority of radiolucent implants.^53^

Regarding proton therapy, no studies have compared the outcomes of different spinal implants. However, multiple retrospective reports on patients with spinal chordomas have consistently indicated a significantly higher rate of local recurrence in patients with titanium implants compared with those without.^54^, ^55^, ^56^ Using multivariate analysis, Snider et al^55^ identified the presence of metallic surgical stabilization as an independent poor prognostic factor (5-year LC rate, with: 46% vs without: 76%, *P* = .01). Based on these reports, many particle therapy facilities do not allow the beam to pass through metal implants.^57^ By contrast, studies on proton therapy with Carbon/PEEK implants suggest that their use reduces various uncertainties.^49^ In addition, a low rate of local recurrence has been reported with Carbon/PEEK implants (recurrence in 1 of 13 patients).^58^

In addition, several studies have reported that the use of Carbon/PEEK implants enables the early detection of recurrence because there are fewer artifacts.^16^^,^^51^^,^^59^ Earlier detection of local recurrence can facilitate modifications in systemic therapies or the pursuit of salvage radiation therapy, while avoiding additional surgical interventions.^51^

However, Carbon/PEEK implants were initially thought to cause increased mid- to long-term fracture risk compared with titanium implants. In a systematic review of 326 patients, the implant-related complication rates during a median follow-up of 13.5 months were 4.7% for the titanium group and 7.8% for the carbon/PEEK group.^12^ Pedicle screw fractures occurred in 2.4% and 1.7% of the respective groups, and the respective reoperation rates were 4.8% and 5.7%, mostly due to implant failure or junctional kyphosis.^12^ A recent large-scale multicenter postoperative cohort study involving 457 patients, the CARBON-S registry, reported favorable surgical outcomes with Carbon/PEEK implants, with an implant-related reoperation rate of only 3% during the follow-up period.^60^ Although these results suggest that the safety profile of Carbon/PEEK implants is comparable with that of titanium, the available clinical evidence remains limited, and further data accumulation is warranted.

### Future perspectives and challenges in clinical implementation

Although Carbon/PEEK implants offer clear advantages over titanium implants in reducing imaging artifacts and improving dose calculation accuracy, several important challenges must be addressed before their routine clinical implementation can be justified. First, although Carbon/PEEK implants significantly reduce imaging artifacts, their direct impact on contouring accuracy in spine SBRT requires further quantitative validation in phantom and clinical studies. Second, although Carbon/PEEK implants are generally expected to provide superior plan robustness in photon therapy because of their radiolucency, this potential advantage should be systematically evaluated using commercially available treatment planning systems. Finally, the most important challenge remains the lack of high-level clinical evidence. Prospective, multi-institutional clinical trials are urgently needed to determine whether the superior imaging and dosimetric properties of Carbon/PEEK implants translate into improved long-term local tumor control and reduced late toxicities in patients.

## Conclusions

This review summarizes the potential benefits of Carbon/PEEK implants compared with titanium implants in spine SBRT. Carbon/PEEK implants exhibit superior physical characteristics with regard to artifacts in CT and MR images, dose perturbations, and dose calculation accuracy and robustness in proton therapy. The reduction of artifacts in postoperative imaging allows optimized radiation treatments, the application of high-dose photon or particle treatment, and the earlier detection of local tumor failure using relatively low-morbidity treatments. However, current evidence remains limited, particularly regarding contouring accuracy across modalities and robustness in photon therapy with different implant materials. Further clinical research is warranted to optimize implant selection criteria and improve the outcomes of postoperative spine SBRT.

## Declaration of AI and AI-Assisted Technologies in the Writing Process

During the preparation of this work the authors used Gemini (Google) in order to edit and refine the English phrasing. After using this tool/service, the authors reviewed and edited the content as needed and take full responsibility for the content of the publication.

## Disclosures

Yuhi Suda received English language editing support for this manuscript from icotec AG. Kei Ito declares receiving payments or honoraria for lectures, presentations, or educational events from Varian Medical Systems. The other authors have no competing interests to disclose.
